# Electron Hopping Across Hemin‐Doped Serum Albumin Mats on Centimeter‐Length Scales

**DOI:** 10.1002/adma.201700810

**Published:** 2017-05-31

**Authors:** Nadav Amdursky, Xuhua Wang, Paul Meredith, D. Jason Riley, David J. Payne, Donal D. C. Bradley, Molly M. Stevens

**Affiliations:** ^1^ Department of Materials Department of Bioengineering and Institute of Biomedical Engineering Imperial College London London SW7 2AZ UK; ^2^ Schulich Faculty of Chemistry Technion – Israel Institute of Technology Haifa 3200003 Israel; ^3^ Department of Physics and Centre for Plastic Electronics Imperial College London London SW7 2AZ UK; ^4^ Department of Physics Swansea University Singleton Park Swansea SA2 8PP Wales UK; ^5^ Department of Materials Imperial College London London SW7 2AZ UK; ^6^ Departments of Engineering Science & Physics Mathematical, Physical and Life Sciences Division University of Oxford Oxford OX1 3PD UK

**Keywords:** current–voltage, electron transfer, impedance spectroscopy, protein films

## Abstract

Exploring long‐range electron transport across protein assemblies is a central interest in both the fundamental research of biological processes and the emerging field of bioelectronics. This work examines the use of serum‐albumin‐based freestanding mats as macroscopic electron mediators in bioelectronic devices. In particular, this study focuses on how doping the protein mat with hemin improves charge‐transport. It is demonstrated that doping can increase conductivity 40‐fold via electron hopping between adjacent hemin molecules, resulting in the highest measured conductance for a protein‐based material yet reported, and transport over centimeter length scales. The use of distance‐dependent AC impedance and DC current–voltage measurements allows the contribution from electron hopping between adjacent hemin molecules to be isolated. Because the hemin‐doped serum albumin mats have both biocompatibility and fabrication simplicity, they should be applicable to a range of bioelectronic devices of varying sizes, configurations, and applications.

Proteins are the main mediators of biological electron transport (ET), the best‐known processes being the mitochondrial respiratory ET chain reaction and the photosynthetic light reaction in plants, algae, and cyanobacteria.^1^ With inspiration from nature, the new field of “bioelectronics”—biologically based and/or biologically targeted electronics—has emerged. In this field, traditional methods with which to measure ET between a donor and acceptor using spectroscopic or electrochemical tools have been replaced by directly contacting the biological material with electrodes and measuring its molecularly bridged conductivity. The most studied biological molecules, melanins, proteins, and DNA are able to mediate electron conduction on the nanometer length scale.^2^, ^3^, ^4^ In addition, it was recently found that the bacterial filament/nanowire can transfer electrons in a molecular electronic junction over micrometers^5^, ^6^ and within bacterial sediments over centimeters (referred to as bacterial cables).^7^, ^8^ The bacterial nanowire is composed of either a filamental protein for the *Geobacter sulfurreducens* bacteria^9^ or by an outer membrane extension for the *Shewanella oneidensis* bacteria,^10^ and in both cases it is decorated by decaheme cytochromes. Electron hopping across multiheme sites in the cytochromes is one of the suggested conductivity mechanisms along the pilus.^11^, ^12^, ^13^ Another proposed mechanism involves metallic‐like conductivity along the backbone of the pilus,^14^, ^15^ and the exact mechanism of conductivity is still under debate.^16^, ^17^, ^18^ The advantageous electron conductivity of the pilus has led to proposals for its use in several applications such as in fuel cells or for remediation.^14^, ^19^ In order to use the bacterial pili as a material for bioelectronics devices or for biomedical application, one should have a purified version of the structural protein together with the cytochromes. However, restrictions for genetically expressing and purifying these proteins in sufficiently large quantities have prevented the opportunity of integrating these proteins in bioelectronic devices.

In this work, we present a new type of freestanding biological‐molecule‐based hybrid material that serves as an effective electron conductor over centimeter‐length scales with enhanced ET relative to the bacterial pilus. We recently demonstrated that electrospun bovine serum albumin (BSA) mats are efficient proton conductors.^20^ One of the major advantages in using BSA mats is the ability of BSA to noncovalently bind a variety of small molecules.^21^ It was previously shown that binding small molecules to proteins (such as BSA) affects their ability to mediate electrons in solid‐state device structures,^22^, ^23^, ^24^ and that tethering ferrocene to redox enzymes can result in an electron mediating relay between the enzyme redox site and the electrode in an electrochemical setup.^25^, ^26^, ^27^ In analogy with solid‐state device nomenclature, this is usually termed a “doping” process, with the selected molecules as “dopants”; one might equally characterize this process as the formation of a hybrid or composite material. Herein, we use the term “molecular doping” for simplicity of acknowledging this fact.

Hemin (an Fe containing porphyrin as shown in **Figure** **1**) was used as the dopant in this work because it is one of the main electron mediators in nature and is also capable, as already noted, of supporting long range ET in the bacterial pilus.^11^, ^12^ Also, it was shown that the electrochemical properties of organic and inorganic films can be improved by mixing them with heme‐containing proteins.^28^, ^29^, ^30^ Figure 1 shows how simply placing a BSA mat in hemin solution for 24 h results in a robust hemin‐to‐BSA binding and a resultant colour change from white to black. UV/Vis absorption was used to quantify the molecular doping of the mat by measuring the absorption of the solution before and after immersing the mat (Figure S1a, Supporting Information). We found an average of 3.93 ± 0.61 nmole of hemin (or equivalently Fe, as there is one Fe atom per hemin) for 1 mm^3^ of BSA mat, which corresponds to 2.37 × 10^18^ atoms of Fe per cm^3^ of mat. The absorption spectrum of the hemin‐doped mats (Figure S1b, Supporting Information) confirms that the oxidation state of the hemins within the mat corresponds to the one in solution. Importantly, while placing the hemin doped mats in a fresh aqueous solution we could not observe any hemin leaching for at least 24 h, indicating that although the BSA is in its unfolded state in the mat, it retains its ability to strongly bind hemin. We used X‐ray photoelectron spectroscopy (XPS) to further study the effect of hemin doping on the BSA electronic structure, and specifically the valence band structure of the protein mat (Figure S2, Supporting Information). It can be seen that in the nondoped mat there are no electronic states present in the gap, above the valence band maximum up to 0 eV binding energy, whereas upon doping we find additional electronic states present in the gap. This might be expected to lead to enhanced conduction, with at least a greatly reduced (or no) energy gap between occupied electron transport levels and the empty energy levels. The XPS survey scans also confirm the presence of Fe in the mats, and enabled us to quantify the amount of remaining salt in the mats upon doping (Figure S3 of the Supporting Information and discussion within).

**Figure 1:**
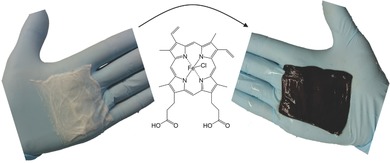
Hemin doping. Images of the nondoped (left) and hemin‐doped (right) BSA mats are shown together with the chemical structure (centre) of hemin.

Recently, we showed that (nondoped) hydrated BSA mats can conduct via proton transfer over millimeter distances with associated conductivity of σ_Η_ ≈ 50 µS cm^−1^.^20^ The large proton conductivity value was attributed to the abundance of oxo‐amino‐acids in the mat, aided by a water‐based hydrogen‐bonded network. We also concluded that bulk proton conduction was the dominant process contributing to the high frequency AC electrochemical impedance spectroscopy (EIS) and DC current–voltage (*I–V*) responses, which exhibited the same distance and temperature dependences. Here we investigate the effect of hemin doping on in‐plane mat conductivity, across larger distances than in our previous study but using an otherwise identical geometry (see Figure S4, Supporting Information). In a similar fashion to the case of the nondoped hydrated mats^20^ here also the measurements were conducted in the water‐swollen state, i.e., at ≈150 wt% hydration. Frequency‐dependent AC EIS measurements are highly informative as they enable deconvolution of charge transport processes having different time constants. While the high‐frequency (≳1 kHz) AC domain is mainly governed by charge carrier bulk transport, the low‐frequency (≲100 Hz) domain is governed by trap‐related carrier capture and release and/or by carrier accumulation next to the contacts due to contact resistances.^31^, ^32^ In DC *I–V* measurements, on the other hand, it is hard to distinguish between these processes.

Unlike our previous study where we used only symmetric Au finger array electrodes,^20^ in this study we use both Au and Ti electrodes, since (as will be discussed below) the chloride ion of the hemin (Figure 1) can electronically interact with the Au electrode. As shown by Bode (absolute impedance as a function of frequency) and Nyquist (imaginary part of the impedance as a function of the real part) plots of the EIS measurements (**Figure** **2**; Figure S5 of the Supporting Information, respectively), the impedance at high‐frequency of the doped mat is around 35–45‐fold lower than that of the nondoped mats (with the same measurement geometry) for all of the measured electrode‐separation distances (Table S1, Supporting Information).^33^ This leads to fitted conductivity values of ≈2 mS cm^−1^ for the doped mats. Accordingly, while the resistance of the nondoped mats allowed us to measure only over distances up to 2.5 mm, the lower resistance of the doped mats allowed us to increase the electrode separation distance up to 24 mm (Figure 2c,d). Importantly, the measured high‐frequency EIS response across the mats was very similar between the two electrode materials (Figure S6, Supporting Information). We conclude that doping the mats with hemin significantly increases the conductance across the mat, which is not expected to be due to an increase in proton conductivity (vide infra) but rather due to electron transport mediated by the hemin moieties. As observed by the XPS results (Figure S3, Supporting Information), the doped BSA mats do contain some sodium, which can be attributed to the presence of ≈30 × 10^−3^ m NaCl in the doping solution. Hence, to support our electron transport hypothesis, and to exclude the possible contribution of ionic conduction of higher mass ions to the measured high conductance across the doped mats, we measured the EIS conductance of nondoped mats that were placed in 50 × 10^−3^ m and 0.5 m NaCl solution (Figure S7, Supporting Information). As shown in Figure S7 of the Supporting Information, placing the nondoped mats in 50 × 10^−3^ m NaCl solution, which is of slightly higher concentration that what was used in the doping solution, resulted in only 20%–40% increase (1.2–1.4‐fold) in conductance for the different measured distances. Furthermore, placing the nondoped mats in 0.5 m NaCl solution induced only a further minor increase in conductance in the order of 50–70% (1.5–1.7‐fold) compared to the nondoped mats in deionized water. These results strongly support our conclusion that the hemin molecules induce the high conductance of the mat. We also measured the EIS across a thin film of only hemin molecules that was formed by drop‐casting a similar hemin solution that was used to dope the mats (Figure S8, Supporting Information). The measured conductance across the drop‐cast hemin film was comparable to the one measured for the hemin‐doped BSA mats, and importantly, the complex shape of the Nyquist plot for the hemin‐doped BSA mats is also present in the drop‐cast hemin films.

**Figure 2:**
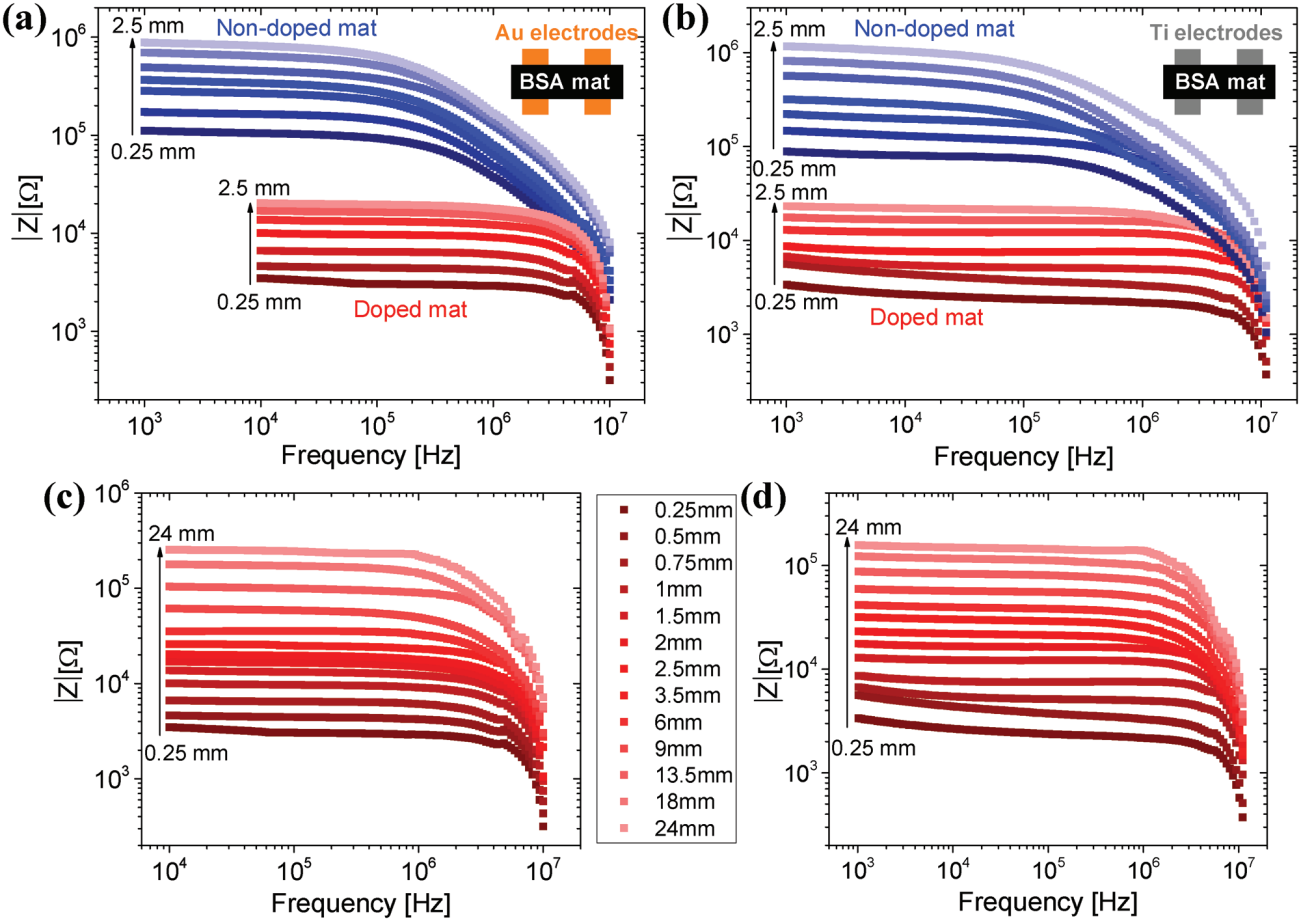
AC impedance response across BSA mats. The absolute impedance as a function of frequency and distance for the nondoped and doped BSA mats measured with a) Au electrodes and b) Ti electrodes. c,d) An extension of the measurements in (a) and (b), respectively, for the doped mats over a larger distances range.

In order to complement the high‐frequency EIS measurements, we compared the DC *I–V* response of the mats before and after doping (**Figure** **3**). It can be clearly seen that measured currents across the nondoped mat are significantly lower than the ones across the doped mats for both the Au and Ti electrodes (Figures S9 and S10 of the Supporting Information compare, pairwise, the *I–V* curves of the nondoped and doped mats for electrode separations in the range 0.25–2.5 mm for the Au and Ti electrodes, respectively). However, while the nondoped mats have monotonically varying *I–V* curves measured with both electrodes, the doped mat exhibited peaks at around ±0.4 V only when measured with the Au electrodes, and a monotonically varying *I–V* curves when measured with the Ti electrodes. The presence of the peaks in the forward and reverse sweeps (Figure S11, Supporting Information), together with the change of the peaks' locations as a function of the distance between the electrodes (Figure S12 of the Supporting Information and text within), leads us to speculate that the observed redox peaks are due to an oxidation and reduction electrochemical process involving the hemin, and specifically the one between the chloride ion of hemin to the Au electrode (since the peaks were observed only by using the Au electrodes).^34^, ^35^, ^36^ Nevertheless, it is important to note that the measured DC conductance (current at low bias) for the doped BSA mats is very similar between the Au and Ti electrodes (**Figure** **4**; Figure S13, Supporting Information), suggesting a similar physical origin. We also followed the DC *I–V* response of the drop‐cast hemin films (Figure S14, Supporting Information), where we found a similar current magnitude to the hemin‐doped BSA mat, but the peaks had a different shape and were located in different positions (≈±0.1 V). The presence of the peaks supports our speculation that the peaks can be associated to the hemin molecule, but more importantly, their different locations imply that the hemin is indeed tightly bound to the BSA scaffold, which changed its electrochemical‐associated potential compared to free hemins in the drop‐cast configuration.

**Figure 3:**
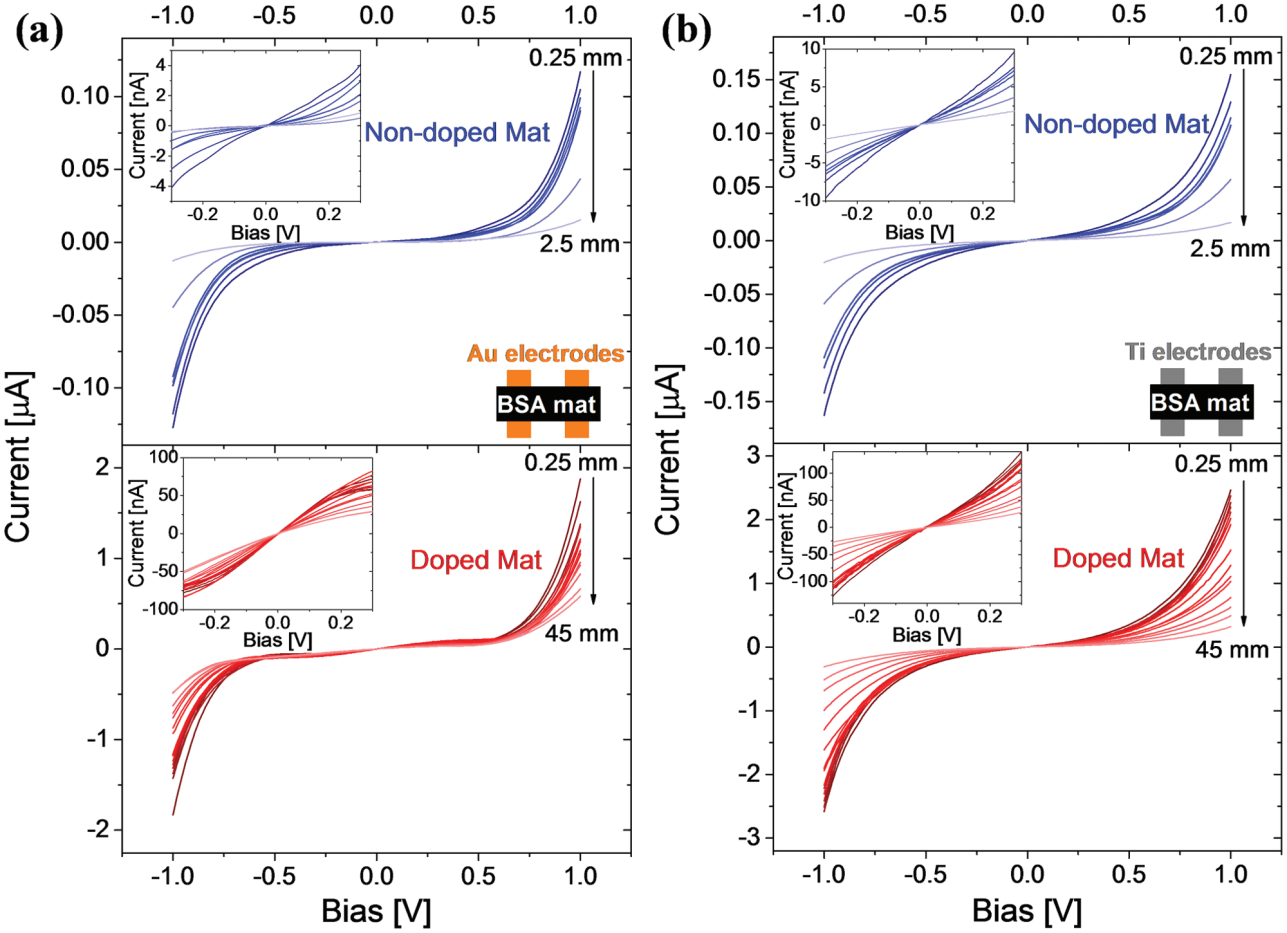
DC current–voltage across BSA mats. *I–V* curves as a function of distance for nondoped (top panels) and doped (bottom panels) mats, measured with a) Au electrodes and b) Ti electrodes. The insets are a magnification of the low‐bias regime.

**Figure 4:**
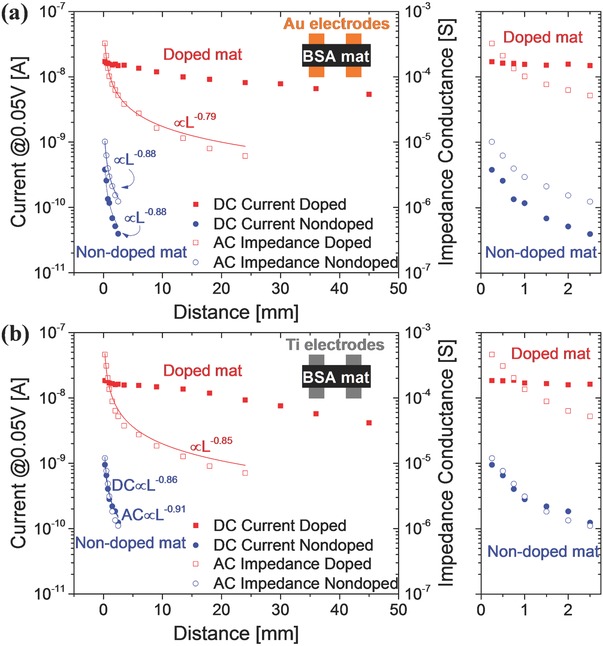
Electrode separation dependence. The interelectrode‐distance dependence of the conductance that was measured with the high‐frequency AC EIS (empty shapes) and low‐bias (at 0.05 V) DC current (filled shapes) for nondoped (circles) and doped (squares) mats measured with a) Au electrodes and b) Ti electrodes. The right panels are magnification of the first 2.5 mm. The lines present power law fits.

The differences between the EIS and *I–V* results for the nondoped and doped mats can be explained by the differences in their charge transport processes. For the nondoped mats, the charge carriers are predominantly protons. The high‐frequency AC EIS measurement is then dominated by bulk proton conduction via hopping through a water mediated network of protein oxo‐amino‐acids.^20^ The nondoped mat DC *I–V* data have the same (bulk proton conduction) origin, with no evidence for deep traps and a DC contact resistance (<10^6^ Ohm) that is negligible in comparison to the bulk resistance (>10^8^ Ohm) (Figure S13a,b, Supporting Information). The similar distance, *L*, dependence of the AC and DC conductance, *G*, which approximately follows Pouillet's law (*G* ∝ *L* ^−1^) supports this perspective (Figure 4, empty and filled circles, respectively). The best fit in both cases gave *G* ∝ *L* ^−*x*^ where 0.86 < *x* < 0.91; the deviation in the exponent from 1 being most probably due to the effects of a finite contact resistance and/or the fractal nature of diffusion across the fibrillar network in the mat.

For the hemin doped mats, charge transport is more complicated. Just as for the nondoped mats, the doped mats are good proton conductors. The two oxo‐acids per hemin molecule (Figure 1) might contribute to this proton conductivity but in comparison to the amount of oxo‐amino‐acids in the protein, and especially compared to the amount of water in the mat, we expect this contribution to be very low, and moreover, due to the bulkiness of the hemin moieties we would expect even a reduction in oxo‐acids volume density. The large decrease in the measured high‐frequency EIS impedance must then be due to the existence of an additional charge transport process, namely ET mediated by the hemin molecules. Metalloporphyrines and metallophthalocyanines are frequently used for the formation of electron conducting molecular semiconductor networks.^37^ The conduction through such molecular networks could be considered as akin to band conduction, which is due to the formation of an ordered crystalline network of the cyclic molecule. By contrast, in our study we “dope” an insulating layer (the BSA mat) with hemin, which binds strongly to the amino‐acid chain of the protein. Hence, we believe we cannot describe the observed ET conduction via a similar band‐like process. Accordingly, we will describe the ET conduction as an intermolecular hopping conduction mechanism (vide infra). In addition to the ET process across the mat, the doped mat also exhibits a significant charge injection (contact resistance) barrier at the interface between the electrodes and the BSA mat (Figure S13c,d, Supporting Information). Since the electronic resistance across the doped BSA mats is significantly lower in comparison to the nondoped mats, the contact resistance (≈3 × 10^6^ Ohm) dominantes the measured DC current, especially for the short distance regime (Figure 4 right panels, filled squares, and insets of Figure S13c,d, Supporting Information). At longer distances, the DC current displays a shallow distance dependency due to the higher resistance of the mat as the distance increases. Accordingly, unlike the high‐frequency EIS measurements of the doped mats that follow Pouillet's law and exhibit a power law decay (Figure 4, empty squares), the DC measurements of the doped mats do not follow bulk ET across the doped mats, i.e., do not exhibit a power law decay as a function of distance (Figure 4, filled squares). A second “interface ET process” takes place only when the doped mat was measured with the Au electrode, which is an electrochemical reaction most probably between the Au electrode and the chloride ion of the hemin molecule. This type of interface ET should not be related to the distance between electrodes. Nevertheless, since the low‐bias DC resistance is very similar between the samples measured with the Au electrodes to the ones measured with the Ti electrodes (Figure S13c,d, Supporting Information) we can conclude that the same type of contact resistance determines the resistance in both cases.

In short, in addition to the proton conductivity measured across nondoped mats, doped mats exhibit both bulk electron conductivity and a high‐resistance interface electron injection. They also yield different temperature dependences (Figure S15, Supporting Information). In our previous study on nondoped mats we demonstrated that both the AC EIS and DC conductivities are thermally activated, with an identical activation energy ≈0.3 eV, as expected for a single proton hopping process. We find, however, for the doped mats that the activation energy for the AC (EIS‐measured) conductance is 0.2 ± 0.04 eV (inset to Figure S15a, Supporting Information), while that for the DC (*I*–*V* measured) conductance is 0.15 ± 0.02 eV (inset to Figure S15b, Supporting Information), both of which are different from that for the nondoped mats.

Given our AC and DC findings above, the bulk electron conductivity of the doped mats can be described using the same model proposed for electron hopping between the decaheme cytochromes along the bacterial pilus.^38^, ^39^ The model is based on the nonadiabatic electron transfer rate (*k*_ET_) equation, also known as the Marcus–Hush formalism:

(1) $$k_{\mathrm{ET}}\;=\;\sqrt{\frac{\pi }{\hbar^{2}\lambda k_{B}T}}\;H_{\mathrm{DA}}^{2}\exp\;\left[-\;\frac{{\left(\lambda \;+\;\Delta G^{0}\right)}^{2}}{4\lambda k_{B}T}\right]$$

where λ is the reorganization energy, *k*_B_ the Boltzmann constant, *T* the absolute temperature, *H*_DA_ the electronic coupling matrix between adjacent (donor and acceptor) molecules, and ∆*G* ^0^ the energetic driving force. Polizzi et al.^38^ thereby derived the interheme D–A distance dependence of the hopping rate (*k*_hop_):

(2) $$k_{hop}\;=\;10^{13}\;\exp\;\left[-\beta \left(r_{nn}\;-\;r_{0}\right)\;-\;\frac{{\left(\lambda \;+\;\Delta G^{0}\right)}^{2}}{4\lambda k_{B}T}\right]$$

where 10^13^ is the maximum charge transfer rate (for direct contact between hemes), β the distance decay constant, *r*_nn_ the intermolecular separation, and *r*_0_ the van der Waals contact distance (0.35 nm).

The conductance of a sample can be described by:

(3) $$G\;=\;\frac{ANe\mu }{L}$$

where *A* is the cross‐sectional area between electrodes separated by distance *L*, *N* the charge carrier density, *e* the electron charge, and μ the charge carrier mobility. The latter can be expressed, via the Einstein relation, as:

(4) $$\mu \;=\;\frac{e}{k_{B}T}\;D$$

where the diffusion constant:

(5) $$D\;=\;k_{\mathrm{hop}}\;r_{\mathrm{nn}}^{2}$$

Taken together, *G* can then be expressed as:

(6) $$G\;=\;\frac{ANe^{2}k_{\mathrm{hop}}r_{\mathrm{nn}}^{2}}{k_{B}TL}$$

Using this model and our EIS‐measured conductance values (which follows bulk ET as discussed above), the values for λ, *r*_nn_, and *k*_hop_ can be estimated for the doped mat. Due to the small deviation of our measured bulk conductance from Pouillet's law (*G* ∝ *L* ^−1^) we have used our measured values (*G* ∝ *L^−^* ^0.79^ and *G* ∝ *L^−^* ^0.85^ for Au and Ti electrodes, respectively, Figure 4) in Equation (6) to yield a better fit to the results. The driving force can be expressed as ∆*G* ^0^ = ∆*G*_total_/*N*, where ∆*G*_total_ is the driving force between the electrodes and *N* is the number of sites. Since a low bias (0.05 V = ∆*G*_total_) was distributed along the numerous hemin sites located between the electrodes, ∆*G* ^0^ is very close to 0 eV. We adopted the β = 1.4 Å^−1^ value calculated by Breur et al. for the distance decay between two heme molecules^40^ and took a charge density value of *N* = 2.37 × 10^18^ per cm^3^ (i.e., the Fe atom density obtained from UV–Vis measurements, see above). We considered a range of reorganization energies, λ = 0.7–1.1 eV, as found in the bacterial decaheme cytochrome.^40^ For each value of λ we calculated the required distance between two adjacent hemin molecules (*r*_nn_) that would match our measured conductance, yield a reasonable hopping rate (*k*_hop_), and be in line with theoretically calculated interheme ET rates (see Figure 2 in ref. 40). Using these constraints, the best fit (*R* ^2^ ≥ 0.98) was found for a *r*_nn_ value of 5.5 Å and λ values of 0.84 and 0.75 eV for the measurements with Au and Ti electrodes, respectively (Table S2). The resulting *k*_hop_ between adjacent hemin molecules then lies between 1.9 × 10^9^ s^−1^ (for λ = 0.84 eV) and 4.3 × 10^9^ s^−1^ (for λ = 0.75 eV). In relation to the theoretical study of Breuer et al.,^40^ our calculated interhemin distances and ET rates suggest that the average spatial orientation of the hemin porphyrin ring planes is either T‐shaped or coplanar (rather than co‐facial). We can use the obtained values for λ to calculate the activation energy of the process $\left(E_{a}\;=\;\frac{{\left(\lambda \;+\;\Delta G^{0}\right)}^{2}}{4\lambda }\right)$ to be 0.19–0.21 eV. These values correlate well to our measured value for the EIS measurements (≈0.2 eV, Figure S15 of the Supporting Information), which further implies that the rate limiting step in the EIS measurements is indeed bulk ET, and not interface ET. It is important to note that since the BSA mats are very complex structures, the different binding sites for hemin in the structure will result in different energetic properties of the hemin (redox potential) and accordingly, the λ, ∆*G* ^0^, and *H*_DA_ will be different between different pairs of adjacent hemin molecules. The values extracted here are set accordingly to be an average for all the different binding sites. Nevertheless, while taking into account the possible wide distribution in the redox potential of the hemin molecules within the BSA mat, in comparison to the Fermi energy of the electrodes, the energy alignment does not restrict the suggested ET mechanism and allows the injection of electrons to the material from the electrodes (Figure S16 of the Supporting Information and text within).

In summary, we have presented a study of the transport properties of hemin‐doped BSA mats that show favourable electron conduction over centimeter distances, hence representing the first of its kind protein‐based conductive free‐standing material that is not being dissolved in an aqueous solution. Furthermore, unlike common conductive organic polymers, where the dopant (usually acids) frequently leaches out of the polymer while placing it in an aqueous solution, our chosen dopant (hemin) has a large affinity to the BSA scaffold and remains within the material in an aqueous solution for months. The resulting conductivity ≈2 mS cm^−1^ is much higher than measured for bacterial pili in a macroscopic configuration (5 µS cm^−1^),^41^ which until now was the only reported protein‐based long range electron conductor. ET in the doped mat is proposed to have a similar origin to one of the mechanisms suggested for the bacterial pili,^38^ namely charge carrier hopping between hemin molecules. However, unlike the evolution optimized system of the bacterial pili, our protein‐based system is completely artificial. Although bacterial pili have been suggested for integration within bioelectronic devices, protein expression of a “clean” system is a very difficult and costly task. By contrast, hemin‐doped BSA mats can be produced much more cheaply, over large areas with significant quantity and in a range of configurations and geometries. Finally, considering both their biocompatibility and ability to sustain cell growth,^42^ hemin‐doped BSA mats appear to represent an excellent ready‐to‐use platform for bioelectronic devices.

## Conflict of Interest

The authors declare no conflict of interest.

## Supporting information

Supplementary
