# Supplementary material for: Electron Hopping Across Hemin‐Doped Serum Albumin Mats on Centimeter‐Length Scales
Source: Adv Mater. 2017 May 31;29(27):1700810. doi: 10.1002/adma.201700810 (PMC5788260; doi:10.1002/adma.201700810)
Supplement: Supplementary file 1 — Supplementary [file ADMA-29-na-s001.pdf]

Copyright WILEY-VCH Verlag GmbH & Co. KGaA, 69469 Weinheim, Germany, 2017.

ADVANCED MATERIALS

Supporting Information

for *Adv. Mater.*, DOI: 10.1002/adma.201700810

Electron Hopping Across Hemin-Doped Serum Albumin Mats on Centimeter-Length Scales

Nadav Amdursky, Xuhua Wang, Paul Meredith, D. Jason Riley, David J. Payne, Donal D. C. Bradley, and Molly M. Stevens**

Copyright WILEY-VCH Verlag GmbH & Co. KGaA, 69469 Weinheim, Germany, 2013.

Supporting Information

Electron Hopping Across Hemin-Doped Serum Albumin Mats on Centimetre-Length Scales

Nadav Amdursky, Xuhua Wang, Paul Meredith, D. Jason Riley, David J. Payne, Donal D. C. Bradley, Molly M. Stevens**

Materials and Methods

Electrospinning of BSA mat – Bovine serum albumin (Sigma-Aldrich, A3608, ≥96%) was used to form the mats. The BSA was dissolved in 90% TFE to a final BSA concentration of 14% (w/v). β-mercaptoethanol was added to the solution to a final concentration of 5% (v/v). A custom built electrospinning system was used, where a bias of 11.5 kV was applied on an 18-gauge blunt needle, and the collector was grounded. The distance between the collector and the end of the needle was 11 cm, and the rate of injection was 0.8 mL/min.

Metal finger-shaped electrode preparation – Several cleaned microscope slides were used as the substrates. An MBraun thermal evaporator inside a nitrogen filled glovebox system under the vacuum of 5x 10^{-7}mbar was used to deposit ~60nm Au thin films and ~55 nm Ti thin films, respectively. Prior to the Au evaporation, a 10nm chromium layer was deposited on the glass substrates as an adhesive layer. A tungsten boat was used for the evaporation of Au, and an alumina coated tungsten boat was used for the evaporation of Ti, since Ti reacts with tungsten in direct contact. The patterning electrodes were defined by a custom made metal mask with varied distances.

Impedance spectroscopy – The impedance spectroscopy was carried out by an SI 1260 impedance/gain-phase analyser (Schlumberger). At least 24 h before the measurement the dry

mats were placed in either deionised water for the non-doped mat, in NaCl solutions of 0.05 M or 0.5 M for the salt-doped mats, or in 0.1 mM hemin (Sigma-Aldrich, ≥97%) solution that was prepared by diluting an aliquot from a stock solution of 12 mM hemin in dimethyl sulfoxide to an aqueous solution (×0.2 PBS buffer, pH7.4, final salt concentration: 27.4 mM NaCl, 2 mM PO_4^{3-} and 0.5 mM KCl, final hemin concentration: 0.15 mM) for the doped mat. Following the incubation of 24 h, the mats were placed in water to wash away any unbound dopants and the wet mats were placed on the finger-shaped electrode (Figure S4) and were dried by a filter paper to remove any excess water molecules that are not tightly bound to the surface. For the measurements with the hemin film, the above dopant solution containing 0.15 mM hemin was drop-casted on the finger-shaped electrode and left to dry. Micromanipulators probes were used in order to contact the gold electrodes. The frequency range was 10 MHz – 1 KHz with an applied AC bias of 50 mV, an integration time of 0.5 s, and no DC bias was applied. The impedance spectra were fitted using the ZView software (© Scribner Associates, Inc).

Current-Voltage measurements – The current-voltage sweeps were carried out by a Keithley 2400 source-measure unit controlled by a computer via a home written Labview program. The same finger-shaped electrodes, mats and micromanipulators probes were also used for the impedance spectroscopy measurement, where one of the probes was biased while the other was grounded.

Temperature dependence – The gold finger-shape electrode with the mat was placed on a heater controlled by a Keithley temperature controller. The impedance and *I-V* measurements were conducted exactly as described above.

X-ray photoelectron spectroscopy (XPS)– The XPS spectra were obtained on a Thermo Fisher K-Alpha utilising a monochromatic Al-Kα X-ray source (hν = 1486.7 eV). Samples were positioned with the electron take-off angle normal to the surface with respect to the analyser. The valence band measurements were measured with a pass

energy of 40 eV, and 0.2 eV step energy, over the range of -5 eV to 40. A low energy electron/ion flood gun was used to ensure an effective surface charge compensation.

UV/Vis absorption – The UV/Vis absorption of the hemin solution was measured by a NanoDrop 2000 spectrophotometer (Thermo Scientific) using a quartz cuvette with a 4 mm pathlength. The absorption spectra were taken before placing the mats and after ~48 h where the mats were in the solution with mild (60 rpm) shaking.

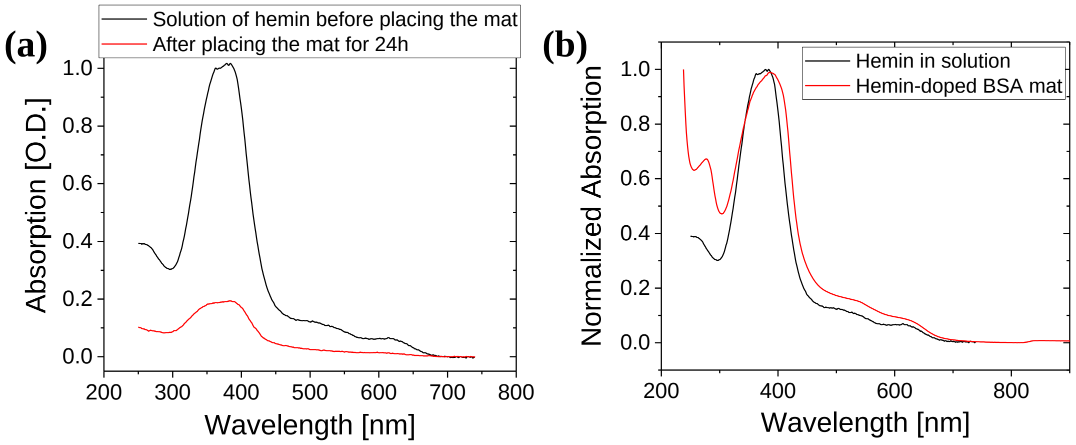

Figure S1. (a) A typical UV-Vis absorption spectra of hemin solution (75 μM) before placing the mat (black curve) and after 24 h where the mat was placed in the solution (red curve). The path length was 4 mm. (b) A normalized absorption comparison between the hemin-doped mat and free hemins in solution. Due to the high absorbance of the BSA mat we used in this experiment a thinner mat (~25 μm) that what was used for the other characterisations together with an integrating sphere.

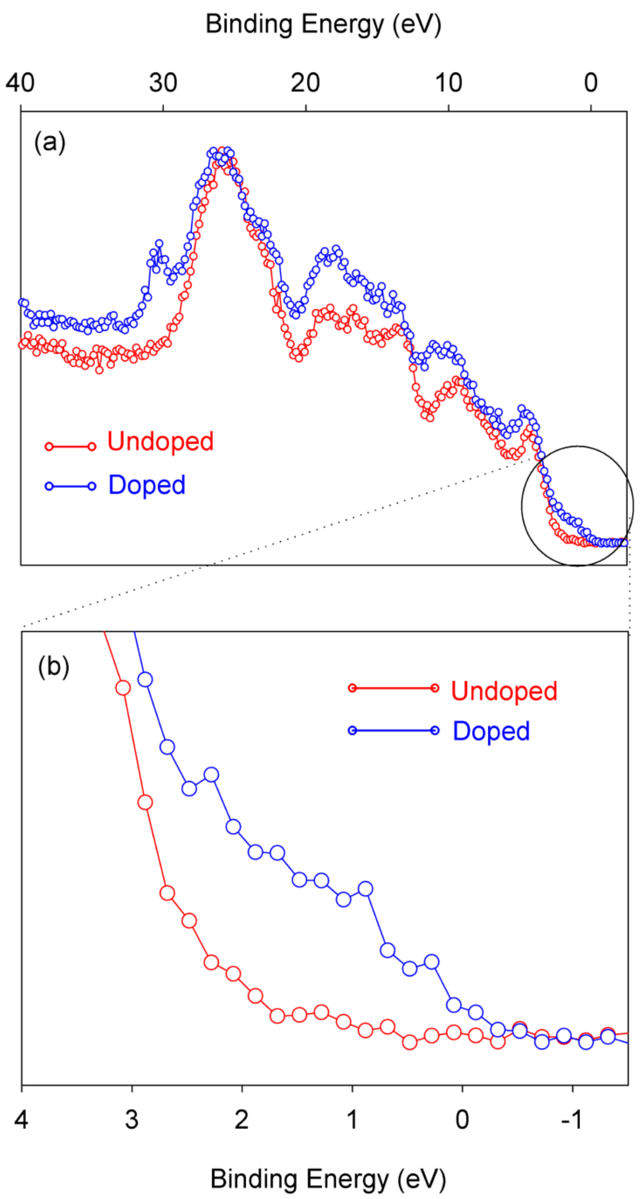

Figure S2. (a) Valence band of the non-doped (red curve) and doped (blue curve) mats. Note the peak at ~30 eV binding energy in the doped sample is due to Na 2*p* states most likely introduced during the doping stage. (b) Expansion of the valence band maximum region showing the increased electronic states above the valence band maximum.

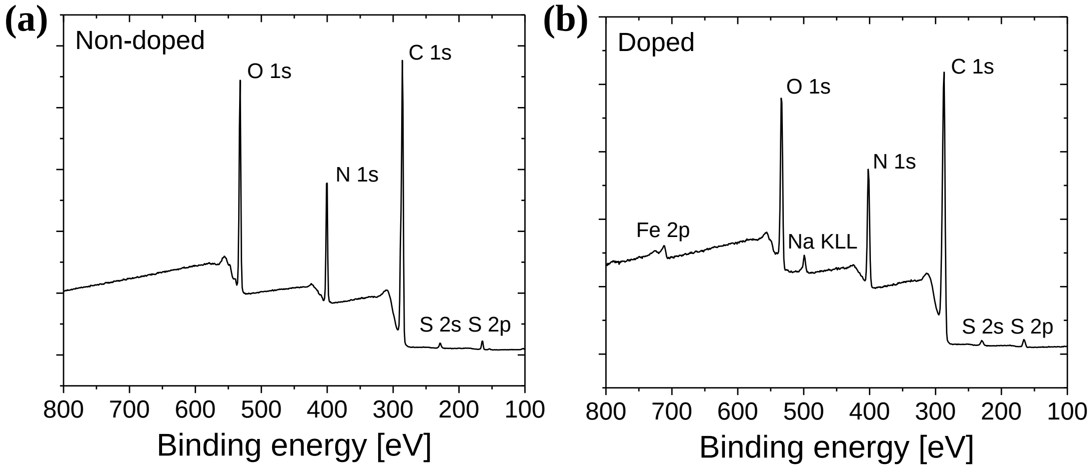

Figure S3. XPS survey scans for the (a) non-doped and (b) doped mats showing the presence of iron in the doped mats as well as a sodium auger peak (Na *KLL*).

The atom ratios from the elemental analysis have been calculated for the non-doped mats. The fair agreement between the calculated ratios to the theoretical values of the atoms in BSA implies that no major impurities have been added to the protein during the electrospinning stage.

	N/S	C/S	O/S	C/N	C/O	O/N
Theoretical values	22.3	84.2	25.7	3.8	3.3	1.2
Measured values	19.2	93.6	25.1	4.8	3.7	1.3

Unfortunately, the low intensity Fe 2*p* peak has not allowed us to extract a quantitative ratio for Fe in comparison to the rest of the atoms, which would have enabled us to estimate the number of hemin molecules per BSA protein.

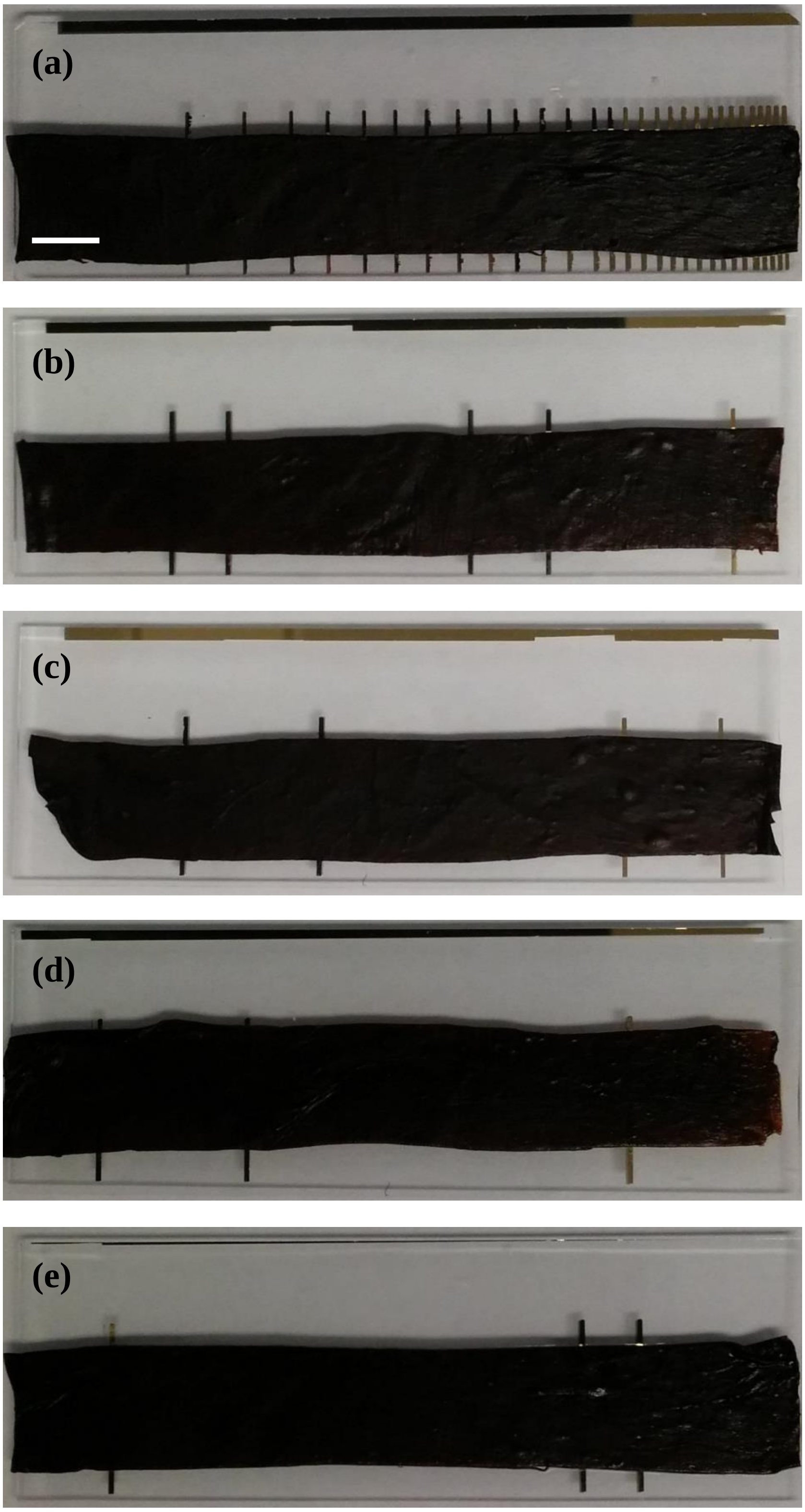

Figure S4. Images of the doped BSA mats on the different Au finger array electrodes. The scale bar represents 5 mm. The distances present in the images are (from left to right): (a) 3.5 – 0.25 mm spacing. (b) 6, 24, 8 (not used in the study), 18 (c) 13.5, 30, 9 mm, (d) 14.5 (not used in the study), 36 mm (e) 45, 6 mm.

Table S1. Resistance values as measured by EIS

Distance [mm]	Non-doped Resistance [Ω]	Doped Resistance [Ω]	Non-doped Conductivity [S·cm^{-1}]	Doped Conductivity [S·cm^{-1}]
		**Au electrodes**		
**0.25**	0.98±0.30·10^5	3.08±0.51·10^3	4.01·10^{-5}	1.28·10^{-3}
**0.5**	1.60±0.39·10^5	4.78±0.65·10^3	4.88·10^{-5}	1.64·10^{-3}
**0.75**	2.52±0.98·10^5	7.40±1.06·10^3	4.65·10^{-5}	1.59·10^{-3}
**1**	3.39±1.35·10^5	9.84±1.10·10^3	4.63·10^{-5}	1.60·10^{-3}
**1.5**	4.72±1.81·10^5	1.30±0.24·10^4	4.97·10^{-5}	1.80·10^{-3}
**2**	6.49±2.73·10^5	1.59±0.47·10^4	4.83·10^{-5}	1.96·10^{-3}
**2.5**	8.08±3.04·10^5	1.92±0.56·10^4	4.86·10^{-5}	2.03·10^{-3}
**3.5**	-	2.61±0.59·10^4	-	2.09·10^{-3}
**6**	-	3.64±0.68·10^4	-	2.58·10^{-3}
**9**	-	6.12±0.81·10^4	-	2.30·10^{-3}
**13.5**	-	8.74±1.05·10^4	-	2.41·10^{-3}
**18**	-	1.25±0.30·10^5	-	2.24·10^{-3}
**24**	-	1.63±0.45·10^5	-	2.30·10^{-3}
		**Ti Electrodes**		
**0.25**	0.83±0.28·10^5	2.17±0.57·10^3	4.67·10^{-5}	1.80·10^{-3}
**0.5**	1.30±0.41·10^5	3.24±0.71·10^3	5.97·10^{-5}	2.41·10^{-3}
**0.75**	2.06±0.85·10^5	4.95±0.96·10^3	5.69·10^{-5}	2.37·10^{-3}
**1**	3.21±1.33·10^5	7.36±1.94·10^3	4.86·10^{-5}	2.12·10^{-3}
**1.5**	5.46±1.88·10^5	1.13±0.21·10^4	4.29·10^{-5}	2.08·10^{-3}
**2**	7.45±2.91·10^5	1.57±0.57·10^4	4.20·10^{-5}	2.00·10^{-3}
**2.5**	9.04±3.59·10^5	1.92±0.70·10^4	4.32·10^{-5}	2.04·10^{-3}
**3.5**	-	2.66±0.89·10^4	-	2.05·10^{-3}
**6**	-	3.63±1.05·10^4	-	2.60·10^{-3}
**9**	-	5.44±1.30·10^4	-	2.58·10^{-3}
**13.5**	-	7.89±1.73·10^4	-	2.67·10^{-3}
**18**	-	1.11±0.32·10^5	-	2.54·10^{-3}
**24**	-	1.41±0.49·10^5	-	2.66·10^{-3}

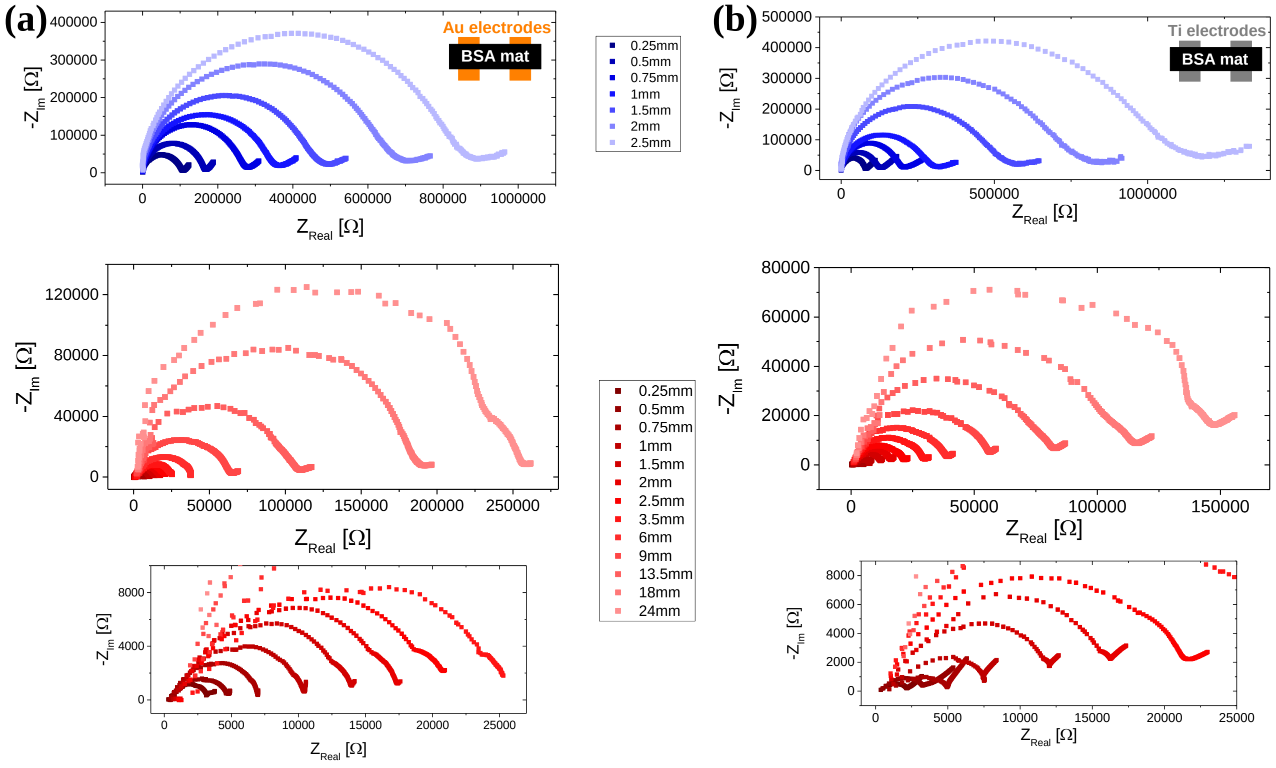

Figure S5. Nyquist plot representation of the imaginary part of the impedance resistance as a function of the real part across non-doped mats (blue squares, upper panels) and doped mats (red squares), measured with (a) Au or (b) Ti electrodes. The lowest panels are a magnification of the low resistance regime of the doped mats displayed in the medium panel. The graphs are plotted on an isometric scale.

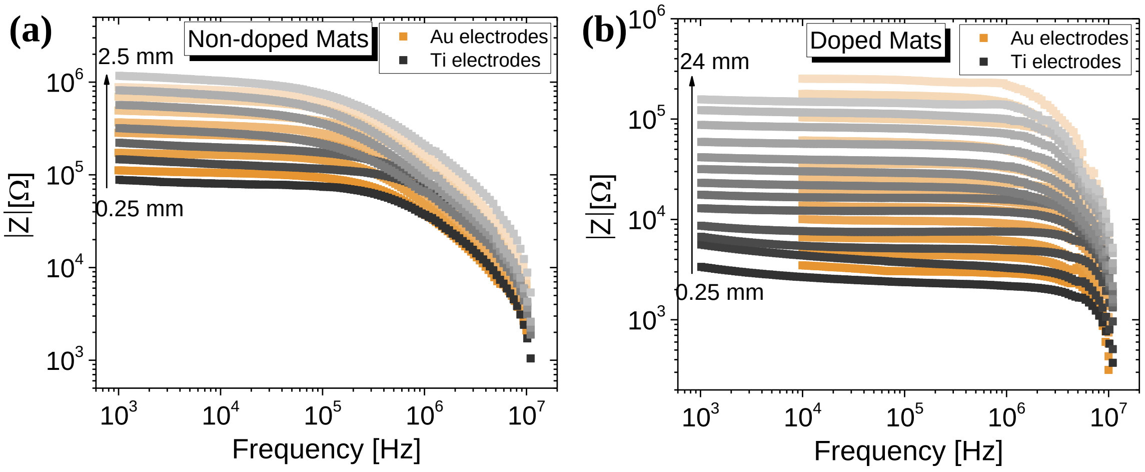

Figure S6. Bode plot comparison of the EIS measurements measured with Au or Ti electrodes for (a) non-doped and (b) doped mats.

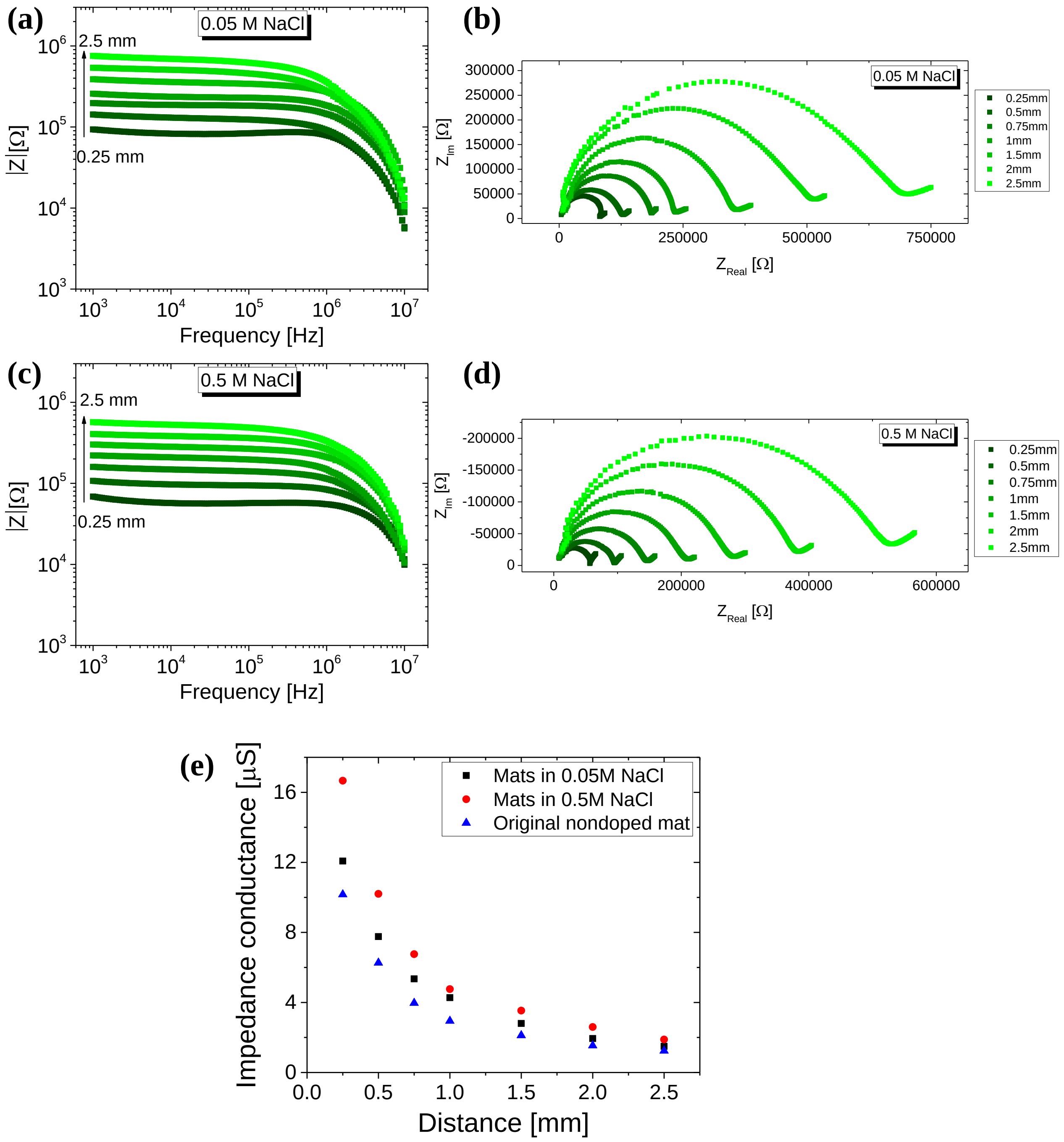

Figure S7. The absolute impedance as a function of frequency and distance for the mats placed in (a) 0.05 M and (c) 0.5 M NaCl. Nyquist plot representation of the imaginary part of the impedance resistance as a function of the real part across the mats placed in (b) 0.05 M and (d) 0.5 M NaCl. (e) The distance dependent conductance across the mats placed in 0.05 and 0.5 M NaCl compared to the mats placed in deionized water.

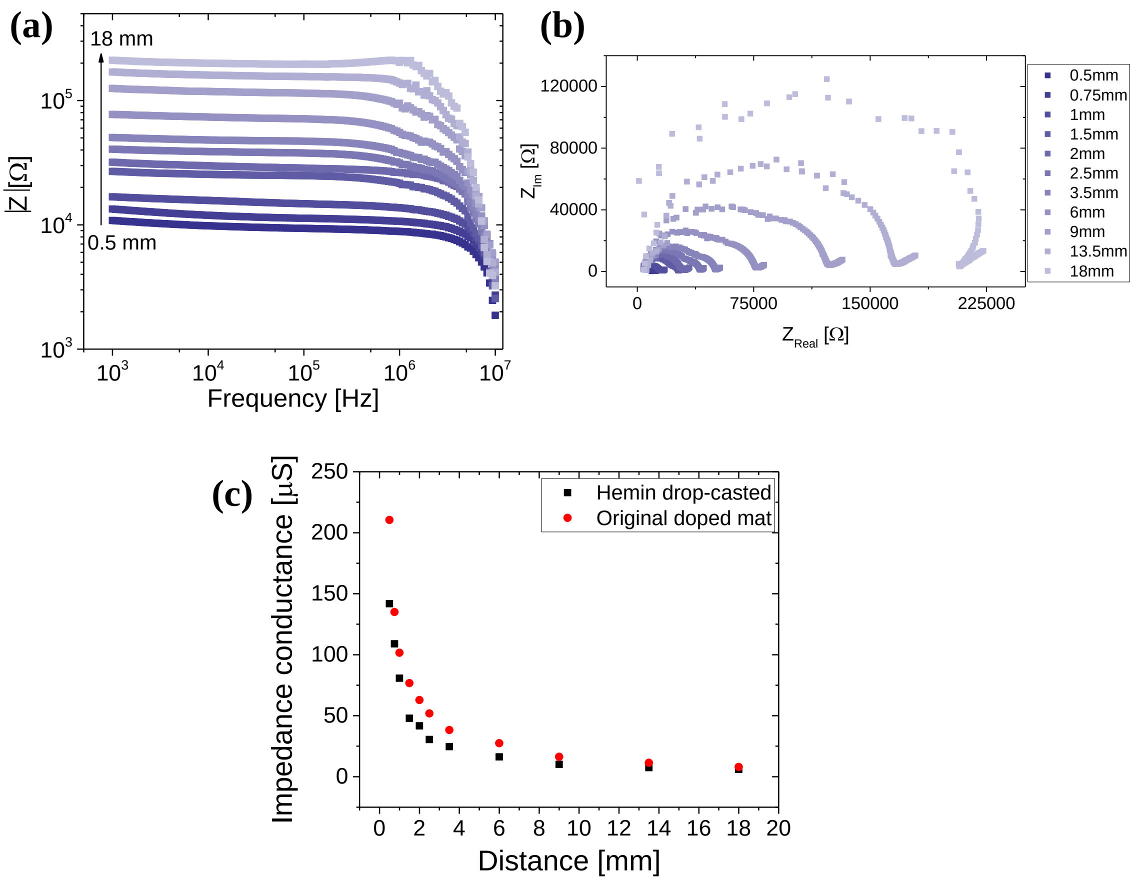

Figure S8. (a) The absolute impedance as a function of frequency and distance together with (b) Nyquist plot representation of the imaginary part of the impedance resistance as a function of the real part across drop-cast hemin films. (c) The distance dependent conductance across the drop-cast hemin films compared to the hemin-doped mats.

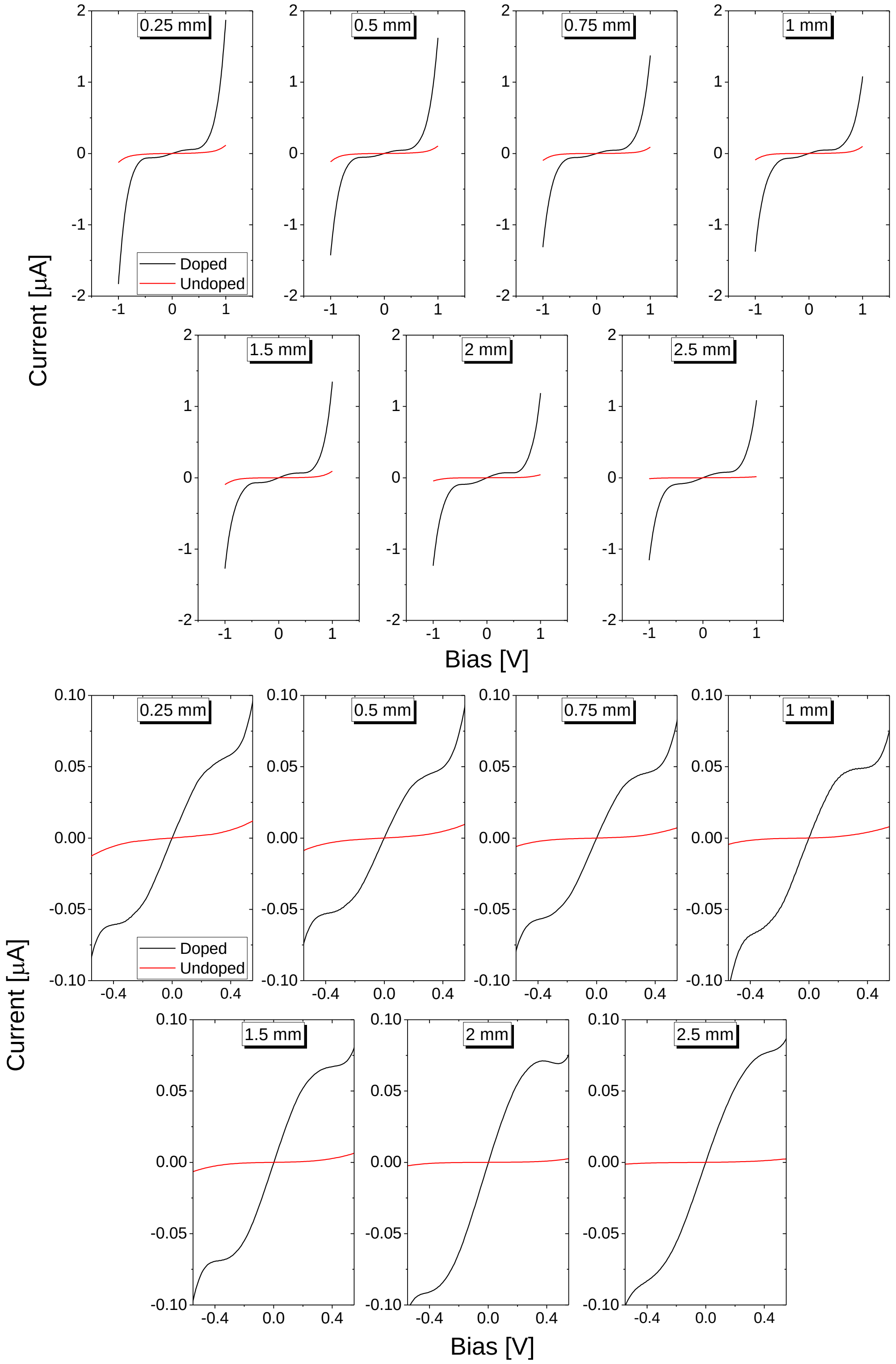

Figure S9. *I-V* comparison between the doped and non-doped mats in the distance range of 0.25 – 2.5 mm measured with Au electrodes. The bottom set of graphs are zoomed areas of the upper set of graphs.

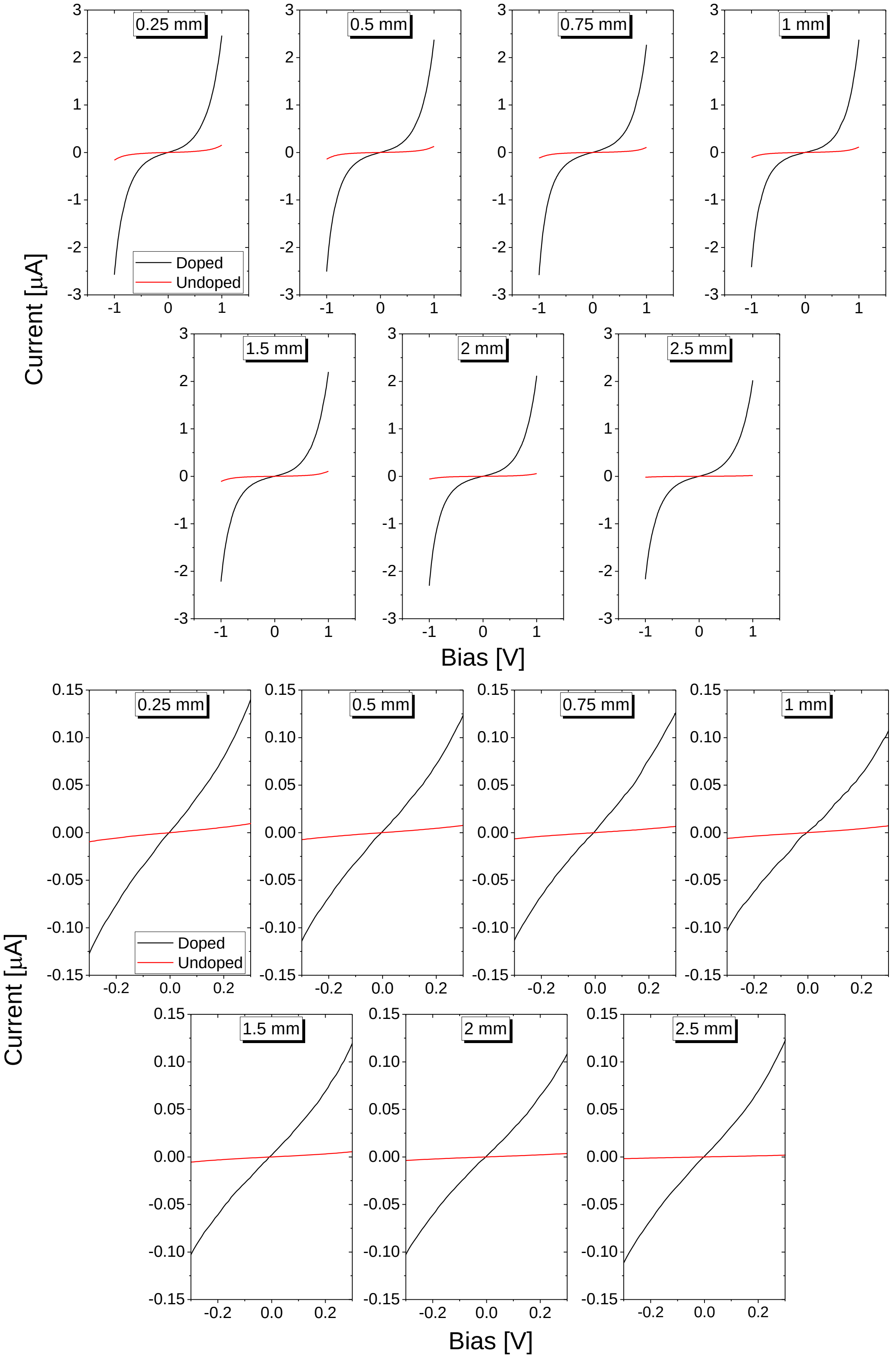

Figure S10. *I-V* comparison between the doped and non-doped mats in the distance range of 0.25 – 2.5 mm measured with Ti electrodes. The bottom set of graphs are zoomed areas of the upper set of graphs.

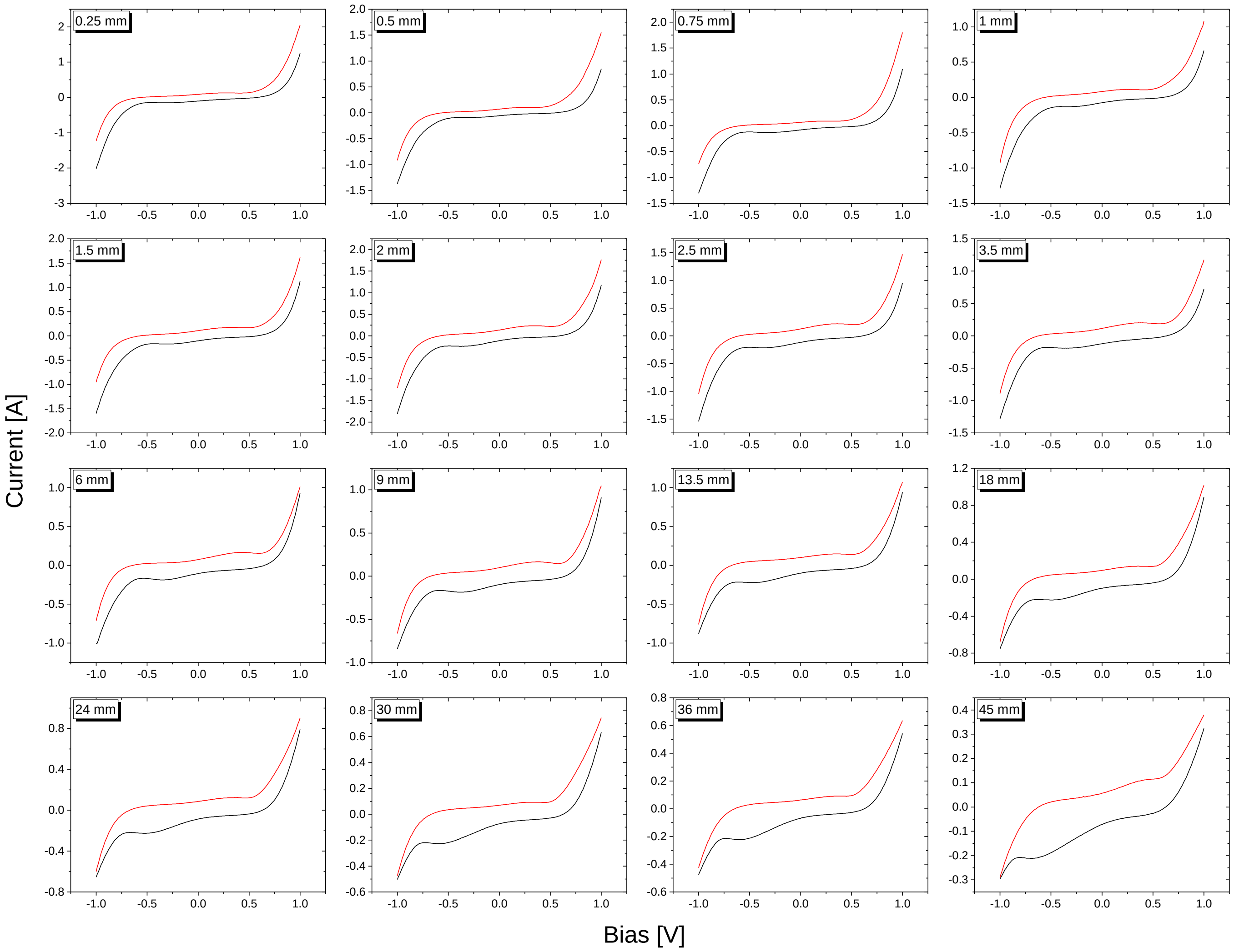

Figure S11. Forward (red curve) and reverse (black curve) *I*-*V* curves across the doped mat on Au electrodes at all measured distances.

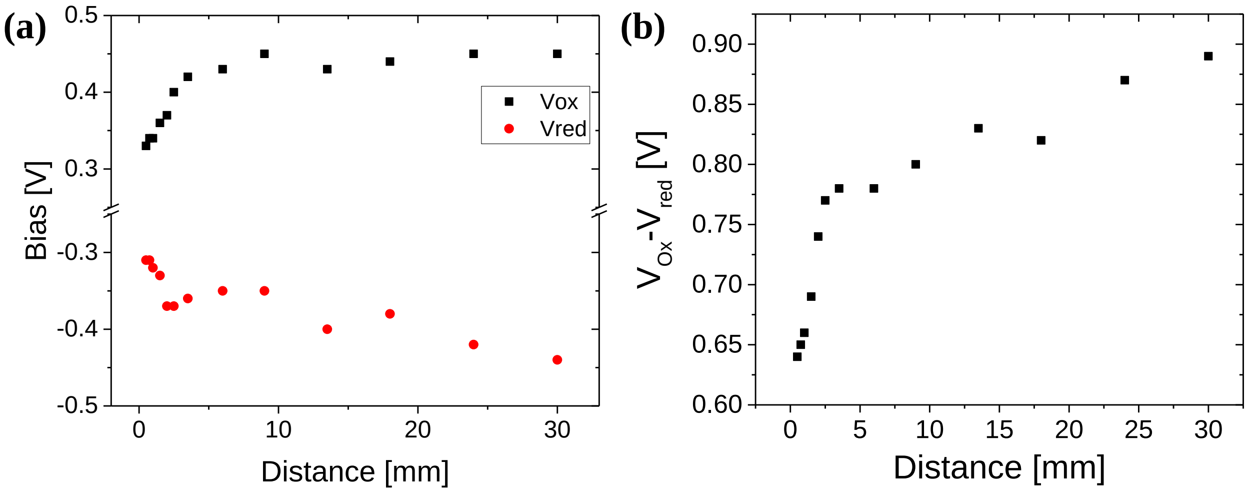

Figure S12. (a) The bias position of the oxidation (V_{ox}) and reduction (V_{red}) peaks and (b) the difference between them as a function of the distance.

The values for the location of the oxidation and reduction peaks for the different distances are as follows:

Distance [mm]	V_{Ox} [V]	V_{Red} [V]
**0.5**	0.33±0.05	-0.31±0.03
**0.75**	0.34±0.04	-0.32±0.03
**1**	0.34±0.05	-0.32±0.03
**1.5**	0.36±0.03	-0.33±0.05
**2**	0.37±0.04	-0.37±0.04
**2.5**	0.41±0.04	-0.37±0.05
**3.5**	0.42±0.05	-0.36±0.04
**6**	0.43±0.07	-0.35±0.06
**9**	0.45±0.06	-0.35±0.06
**13.5**	0.43±0.06	-0.40±0.07
**18**	0.44±0.07	-0.38±0.06
**24**	0.45±0.05	-0.42±0.08
**30**	0.45±0.07	-0.44±0.07

Since we used the same sweep rate and integration time for the DC measurements at different distances, the longer the distance the faster the electric field was. Accordingly, as in common electrochemical type of measurement, an increase in the rate of the electric field induces an increase in the voltage difference between the oxidation and reduction peaks.

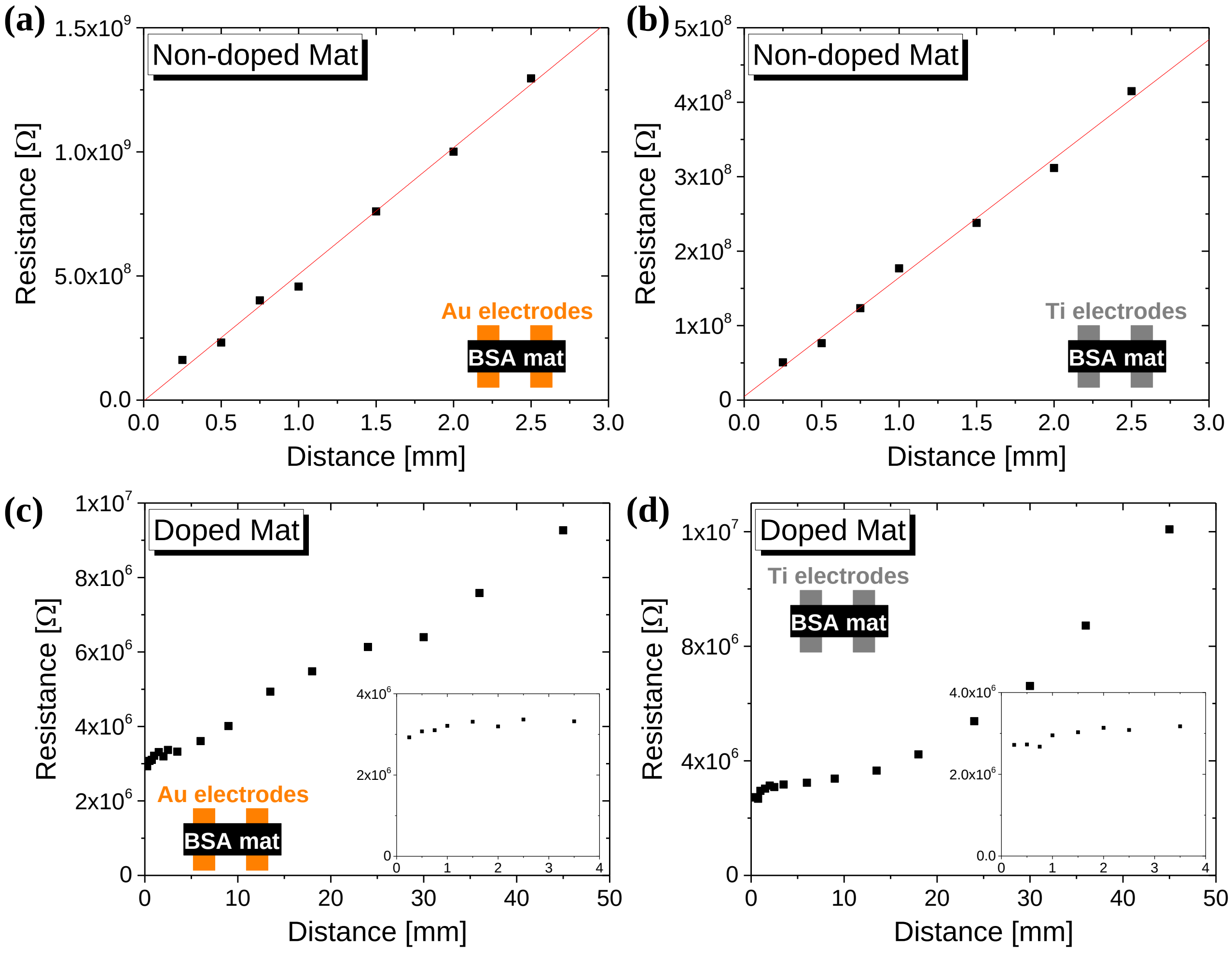

Figure S13. The DC contact resistance estimation for the non-doped (a and b) and doped (c and d) mats, measured with Au (a and c) and Ti (b and d) electrodes. The contact resistance was estimated by the extrapolation of the resistance (extrapolated by a linear fitting to the ±0.05V regime of the *I*-*V* plot) for zero distance. The insets in the lower panels are a magnification of the short distance regime.

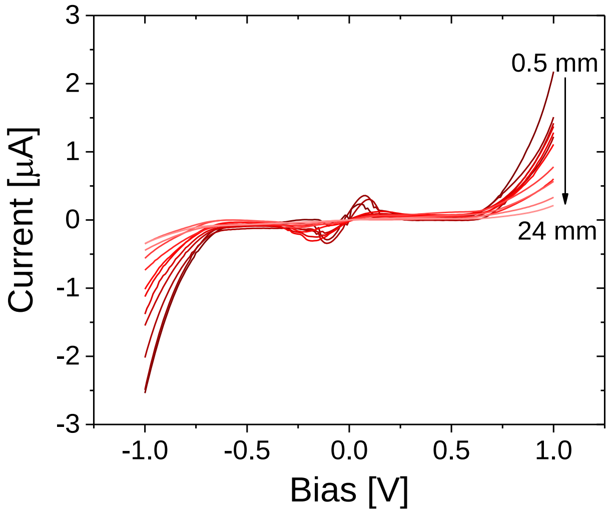

Figure S14. *I*-*V* curves as a function of distance for drop-cast hemin films measured with Au electrodes.

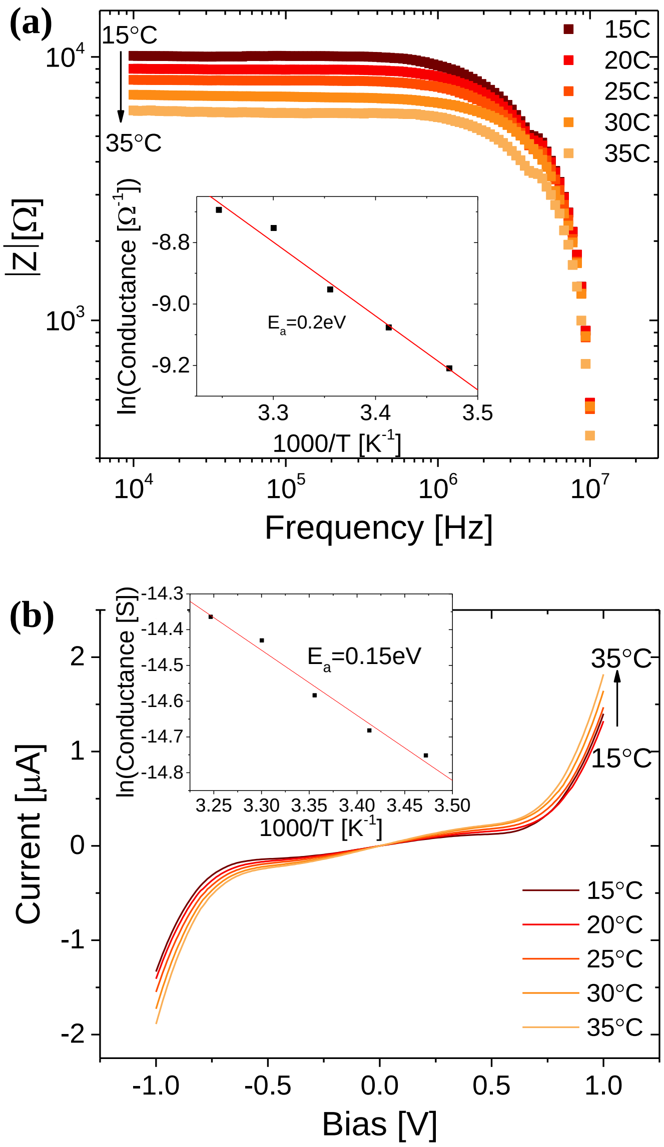

Figure S15. Temperature-dependence of (a) the AC EIS and (b) DC *I*-*V*, taken with the Au electrodes across the 2.5 mm junction. The insets show the activation energy as calculated by fitting the measured conductance/current to an Arrhenius equation.

Table S2. Conductance (G) values obtained by Eq. (4) in the main text ($G = \frac{ACe^2 k_{hop} r_{nn}^2}{k_B T L}$) in comparison to the measured EIS conductance with Au and Ti electrodes. The resulted parameters for the measurements with Au electrodes are: the distance between adjacent hemin molecules is r_{nn} = 5.5 Å, the reorganization energy is λ = 0.84 eV, and the electron hopping rate is k_{hop} = 1.9 × 10^9 s^{-1}. The resulted parameters for the measurements with Ti electrodes are: r_{nn} = 5.5 Å, λ = 0.75 eV and k_{hop} = 4.3 × 10^9 s^{-1}.

**Distance [mm]**	**Measured G – Au [S]**	**Calculated G - Au [S]** R^2=0.99	**Measured G – Ti [S]**	**Calculated G - Ti [S]** R^2=0.98
**0.25**	$3.25 \cdot 10^{-4}$	$3.17 \cdot 10^{-4}$	$4.61 \cdot 10^{-4}$	$4.32 \cdot 10^{-4}$
**0.5**	$2.10 \cdot 10^{-4}$	$1.84 \cdot 10^{-4}$	$3.08 \cdot 10^{-4}$	$2.40 \cdot 10^{-4}$
**0.75**	$1.35 \cdot 10^{-4}$	$1.33 \cdot 10^{-4}$	$2.02 \cdot 10^{-4}$	$1.70 \cdot 10^{-4}$
**1**	$1.01 \cdot 10^{-4}$	$1.06 \cdot 10^{-4}$	$1.36 \cdot 10^{-4}$	$1.33 \cdot 10^{-4}$
**1.5**	$7.67 \cdot 10^{-5}$	$7.71 \cdot 10^{-5}$	$8.86 \cdot 10^{-5}$	$9.42 \cdot 10^{-5}$
**2**	$6.28 \cdot 10^{-5}$	$6.14 \cdot 10^{-5}$	$6.34 \cdot 10^{-5}$	$7.37 \cdot 10^{-5}$
**2.5**	$5.19 \cdot 10^{-5}$	$5.15 \cdot 10^{-5}$	$5.22 \cdot 10^{-5}$	$6.10 \cdot 10^{-5}$
**3.5**	$3.82 \cdot 10^{-5}$	$3.95 \cdot 10^{-5}$	$3.75 \cdot 10^{-5}$	$4.58 \cdot 10^{-5}$
**6**	$2.75 \cdot 10^{-5}$	$2.58 \cdot 10^{-5}$	$2.75 \cdot 10^{-5}$	$2.90 \cdot 10^{-5}$
**9**	$1.63 \cdot 10^{-5}$	$1.87 \cdot 10^{-5}$	$1.84 \cdot 10^{-5}$	$2.05 \cdot 10^{-5}$
**13.5**	$1.14 \cdot 10^{-5}$	$1.36 \cdot 10^{-5}$	$1.27 \cdot 10^{-5}$	$1.45 \cdot 10^{-5}$
**18**	$7.97 \cdot 10^{-6}$	$1.08 \cdot 10^{-5}$	$9.01 \cdot 10^{-6}$	$1.14 \cdot 10^{-5}$
**24**	$6.15 \cdot 10^{-6}$	$8.62 \cdot 10^{-6}$	$7.09 \cdot 10^{-6}$	$8.92 \cdot 10^{-6}$

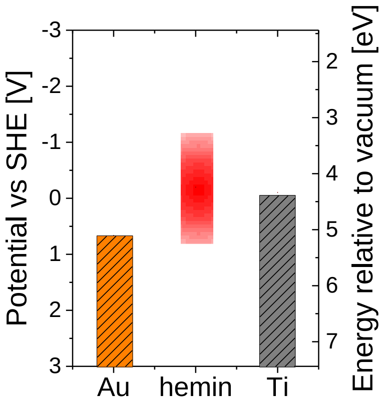

Figure S16. Band alignment of the Au and Ti electrodes around a possible distribution of the hemin molecules within the BSA mat. The energy distribution of the hemin molecules was estimated by a ±1V range around the redox potential of hemin in aqueous solution (~ (-0.1) – (-0.2)V* Vs SHE). Due to the uncertainty in the position of the redox potential, and the band gap of each of the hemin molecules, the exact position of the HOMO and LUMO states are unknown. As suggested from the valence band position in Figure S2, the band gap of the system might be very small, and accordingly the HOMO and LUMO band would be situated close (<~0.5eV) from the redox potential.

*O. S. Ksenzhek, S. A. Petrova, *Bioelectrochemistry and Bioenergetics* **1978**, *5*, 661
